# Automated Analysis for MR Coil QA

**DOI:** 10.1002/acm2.70624

**Published:** 2026-05-13

**Authors:** Bhudatt Paliwal, Aviral Bal, Lokesh Kodali, Dinesh Tewatia, Idarto Tanumihardjo

**Affiliations:** ^1^ Department of Human Oncology University of Wisconsin Madison Madison Wisconsin USA

**Keywords:** automation, magnetic resonance imaging, quality assurance, workflow optimization

## Abstract

**Background:**

Phantom‐based quality assurance (QA) of magnetic resonance imaging (MRI) coils is a standard practice for assessing signal‐to‐noise ratio (SNR), image uniformity, and magnetic field homogeneity. Manual slice selection and region‐of‐interest (ROI) placements introduce operator‐dependent variability and workflow inefficiencies.

**Purpose:**

To develop a unified, fully automated desktop‐based workflow for MRI coil QA that eliminates manual slice selection and ROI placement.

**Materials and methods:**

A Python desktop app was built to automate MRI coil QA workflows for body, torso, and head‐and‐neck coils. It takes DICOM image sets and raw free‐induction decay data and performs slice selection, phantom detection, and fixed‐geometry ROI placement automatically to calculate standard QA metrices: SNR, percent image uniformity, and frequency‐domain magnetic field homogeneity. Thus, obtained results are directly saved in structured reports and plots.

**Results:**

The automated workflow carried out all implemented QA tasks, without manual ROI placement, and consistently produced QA metrices for body, torso, head‐and‐neck, weekly QA, and magnetic field homogeneity analyses. The standardization of the outputs facilitated an objective evaluation of the coils and channel performance by eliminating the subjective bias.

**Conclusions:**

This study presents an automated MRI quality assurance workflow that operates through standardized testing methods and delivers consistent results while decreasing the impact of human error in standard clinical quality control procedures.

## 1 INTRODUCTION

MRI coil quality assurance (QA) is an important part of clinical MRI. It ensures coils are properly functioning for clinical use.^1^ Phantom‐based measurements are often used to measure imaging quality parameters such as signal‐to‐noise ratio (SNR) and percent image uniformity (PIU).^2^, ^3^ Such measures are often based on manual slice selection and region‐of‐interest (ROI) location, introducing operator‐dependent variability that can obscure subtle changes in system performance over time.^1^, ^4^

Most modern MRI quality assurance processes tend to rely on software provided by the vendors, spreadsheet analyses, or custom scripts.^5^ While vendor‐provided QA software can produce reliable results for individual tests, the complete clinical QA workflow for MR‐linac systems such as the ViewRay MRIdian often requires performing multiple separate analyses across different coil configurations. In our clinical practice, this involved combining vendor on‐console analyses with separate spreadsheet‐based data recording and manual result transcription, introducing operator‐dependent variability in the data handling and reporting steps rather than in the analysis algorithms themselves. The proposed application consolidates these separate workflows into a single unified platform that automates the end to end process from DICOM ingestion through structured report generation.

**FIGURE 1:**
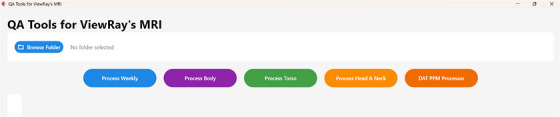
User interface for the automated MRI coil quality assurance application.

This Technical Note proposes a fully automated desktop MRI coil quality assurance approach that integrates multiple phantom‐based approaches to quality assurance into a standard processing pipeline to address these limitations. The automation of workflow consists of dataset import, slice selection, phantom identification, placement of ROI, computation of metrices, and report production. An image‐based quality assurance of weekly checks of body, torso, and head‐and‐neck coils and frequency‐domain analysis of the homogeneity of magnetic fields is combined into one application. The script for applications is written in Python and packaged as a standalone executable, requiring no preinstalled analysis of environments or programming skills. Figure 1 provides an overview of the application interface and the QA workflows.

## 2 MATERIALS AND METHODS

The MRI coil quality assurance measurements have been conducted based on fully automated desktop applications, developed in Python, to process image‐based QA datasets and raw acquisition data. Image‐based processes were performed in accordance with the NEMA‐recommended MRI coil QA protocols.^6^ DICOM datasets were ingested and validated by careful inspection of metadata. Pixel values were transformed into floating‐point arrays, and rescaled slope and intercept parameters were applied. The system evaluated in this study is a ViewRay MRIdian 0.35T MR‐linac system. The acquisition protocols follow the vendor's System Acceptance Test (SAT‐04) procedures. Weekly QA protocols consist of a single‐slice T2‐weighted SSFP acquisition using the body phantom (PN 62505 or 70340) in a transverse orientation. Body coil NEMA protocols involve six separate acquisitions of the NEMA body phantom, capturing signal and noise images in transverse, sagittal, and coronal orientations with Prescan Normalize OFF. The 12‐element torso coil protocols utilize combined‐view acquisitions with Pre‐scan Normalize ON for uniformity and OFF for SNR, plus 12 individual element acquisitions labeled VAS1–VAS3, VPS1–VPS3, VAP1–VAP3, and VPP1–VPP3. The 10‐element head‐and‐neck coil protocols follow a similar combined‐view structure, plus 10 individual element acquisitions labeled VAS1–VAS3, VPS1–VPS3, VAP1–VAP2, and VPP1–VPP2. Finally, magnetic field homogeneity protocols utilize raw free‐induction decay data acquired at multiple gantry angles from 0 degrees through 330 degrees in 30‐degree increments.

When multi‐slice DICOM series are provided, a representative slice is automatically selected based on the highest mean phantom signal intensity at a slice location closest to the isocenter. Automated image segmentation using Otsu thresholding^7^ followed by morphological refinement is used to localize the phantom. Noise ROIs were defined as circular regions with a fixed area of 340 cm^2^ (34,000 mm^2^), centered at the geometric center of the image. The radius in pixels was computed from the desired area using *r* = √(A/π) and converted to pixel units via the DICOM Pixel Spacing metadata. This geometry was held constantly across all datasets to ensure that noise standard deviation measurements were computed over an identical spatial extent, regardless of image resolution or phantom size. For signal ROIs in the combined coil views, a circular ROI of 340 cm^2^ was placed within the automatically detected phantom boundary. For individual coil element analysis, a smaller signal ROI with a 3 mm radius was centered on the pixel of maximum intensity within the phantom. Standard QA parameters, such as SNR and PIU, were computed at routine QA procedures of body, torso, and head‐and‐neck coil workflows. The magnetic field homogeneity of frequency domain data was determined based on the raw free‐induction decay data, which automatically provided center frequency offset and spectral linewidth values in parts per million. The workflow analysis is fully automated, initially with the ingestion of data into the analysis, to generate reports, and the results were exported as structured spreadsheets and plots to enable longitudinal QA tracking. The software tool is not publicly distributed at this time; however, the authors are willing to provide access to the application upon reasonable request for research and evaluation purposes.

## 3 RESULTS

**FIGURE 2:**
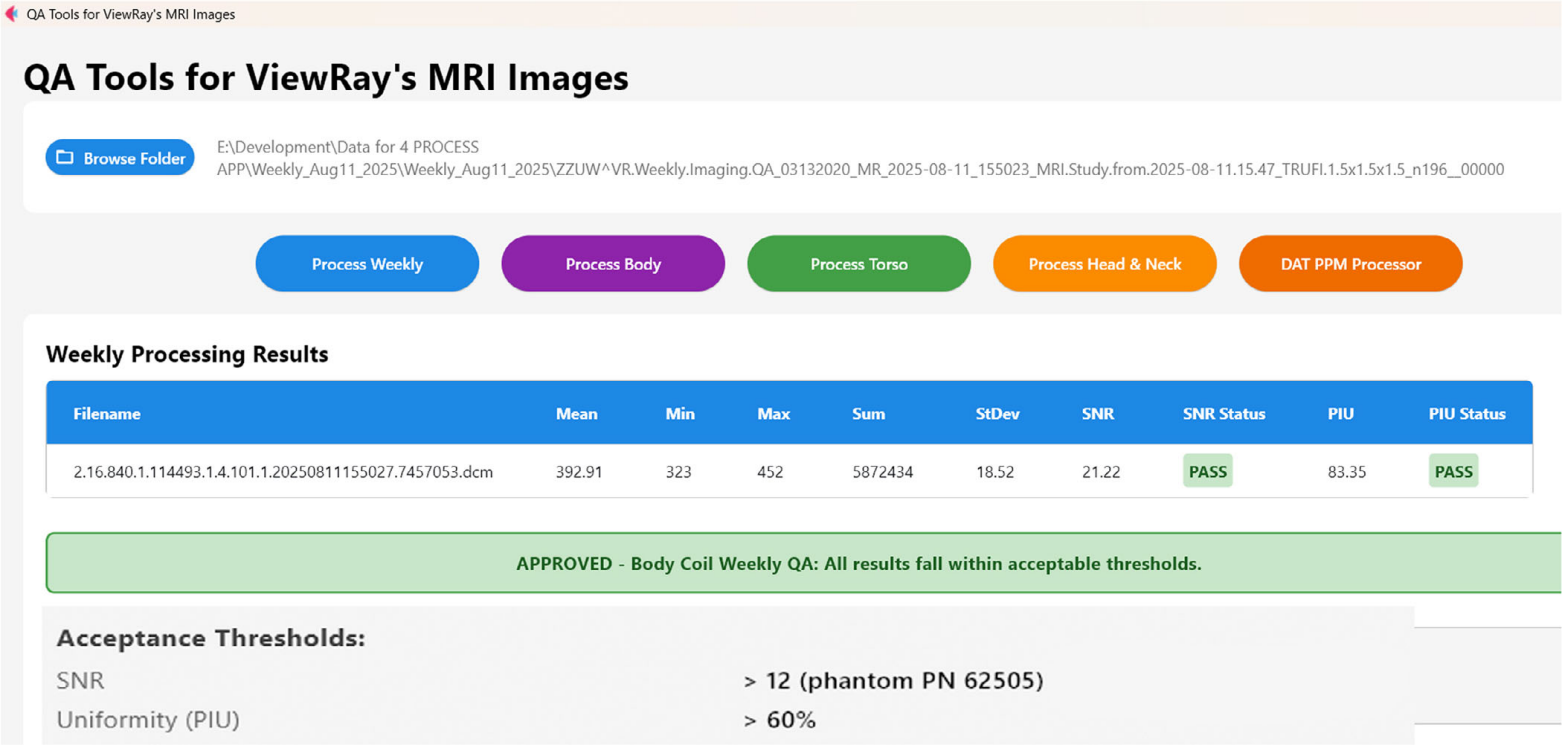
User interface of the automated MRI coil quality assurance application, illustrating the weekly processing workflows.

Our automated workflows efficiently process the MRI quality assurance datasets through all the image‐based methods. It included routine QA and NEMA‐based analyses for body, torso, and head‐and‐neck coils. For each dataset, the steps of slice selection, phantom localization, and region of interest placement were done automatically upon data ingestion. Continuously processing the same dataset led to the same quantitative results, confirming that the extraction of metrics was deterministic and reproducible with no influence from user interaction.^8^, ^9^ The application interface and an example of one of the different processing workflows that can be followed are presented in Figure 2. For every workflow, quantitative QA metrics were automatically extracted and recorded in tabular summaries, including acceptance thresholds, allowing effortless comparison between QA sessions.

Automated extraction of signal statistics, SNR, and PIU were performed from standardized regions for all image‐based QA workflows. The output metrices were formatted in a tabular format that facilitated straightforward comparison across sessions. Table 1 shows examples of automated routine QA results.

**TABLE 1:** Summary of signal statistics, signal‐to‐noise ratio (SNR), and percent image uniformity (PIU) obtained from automated weekly MRI coil quality assurance analysis.

Filename	Mean	Min	Max	Sum	Standard Deviation	SNR	SNR Threshold	SNR Status	PIU	PIU Threshold	PIU Status
x.dcm	392.91	323	452	5872434	18.52	21.2	>11	PASS	83.3	>60	PASS

**FIGURE 3:**
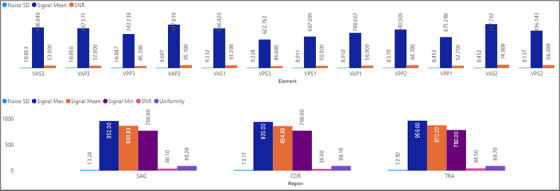
Orientation‐dependent and element‐level signal‐to‐noise ratio results for the torso coil obtained from automated analysis.

**FIGURE 4:**
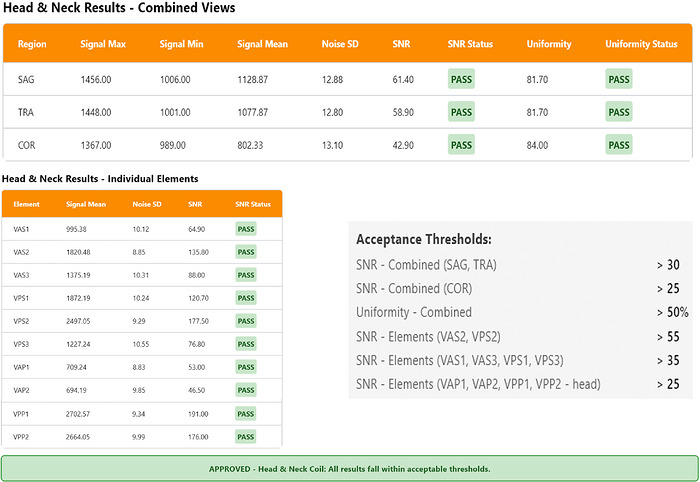
Combined‐view and element‐level signal‐to‐noise ratio and image uniformity results for the head‐and‐neck coil obtained from automated analysis.

The automated workflow generated quantitative results in combined coil views and in single coil elements in the analyses of torso and head‐and‐neck coils. The naming of individual elements is of the vendor naming convention: VAS (Ventral Anterior Superior), VPS (Ventral Posterior Superior), VAP (Ventral Anterior Posterior), and VPP (Ventral Posterior Posterior). The SNR and image uniformity were determined in the sagittal, coronal, and transverse planes enabling evaluation of the overall coil performance as well as the variation at the element level. The software compares these metrics to vendor set System Acceptance Test levels. As an example, combined‐view SNR must be more than 30 at sagittal and transverse orientations and at least 25 at coronal orientations. Individually the anterior elements VAS2 and VPS2 should have SNR > 55, the rest of the standard elements SNR > 35, and finally the posterior head elements VAP1, VAP2, VPP1 and VPP2 SNR > 25. In Figures 3 and 4, an innate tendency of the posterior elements is to have a lower mean signal because it is more distant than the phantom but, nevertheless, it does not pose a threat of crossing the > 25 SNR marker. When the SNR of any element or the overall uniformity of it drops below its individual threshold, the software alerts it as a failure, and a standard clinical policy is then activated to inform the vendor service team about the issue of additional assessment or coil replacement.

### 3.1 Magnetic Field Homogeneity

**FIGURE 5:**
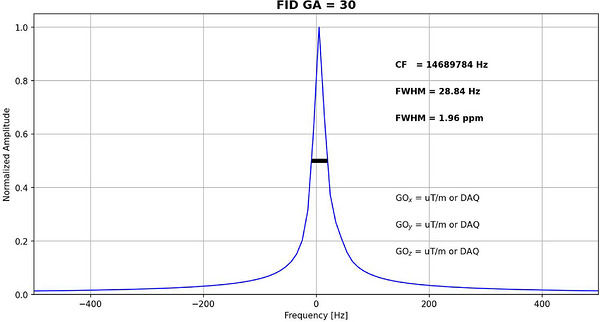
Normalized frequency‐domain spectrum for FID at a gantry angle of 30°, showing the central frequency (CF) and full width at half maximum (FWHM) in Hz and ppm.

Frequency domain analysis yielded normalized spectra corresponding to each gantry angle, each showing the main resonance peak very close to the operating frequency. The central frequency (CF) and full width at half maximum (FWHM) were automatically extracted from each spectrum and served as quantitative indicators of magnetic field uniformity. These parameters enabled systematic comparison of frequency stability and spectral linewidth for different gantry angles.^10^, ^11^, ^12^ The spectral outputs that had been standardized allowed a uniform evaluation across different datasets while remaining sensitive to the variations of the field. Figure 5 displays a typical normalized spectrum, which was recorded at a gantry angle of 30°, where the CF and FWHM determined automatically are also shown, and the consistency of the graphical and tabulated outputs is demonstrated.

**FIGURE 6:**
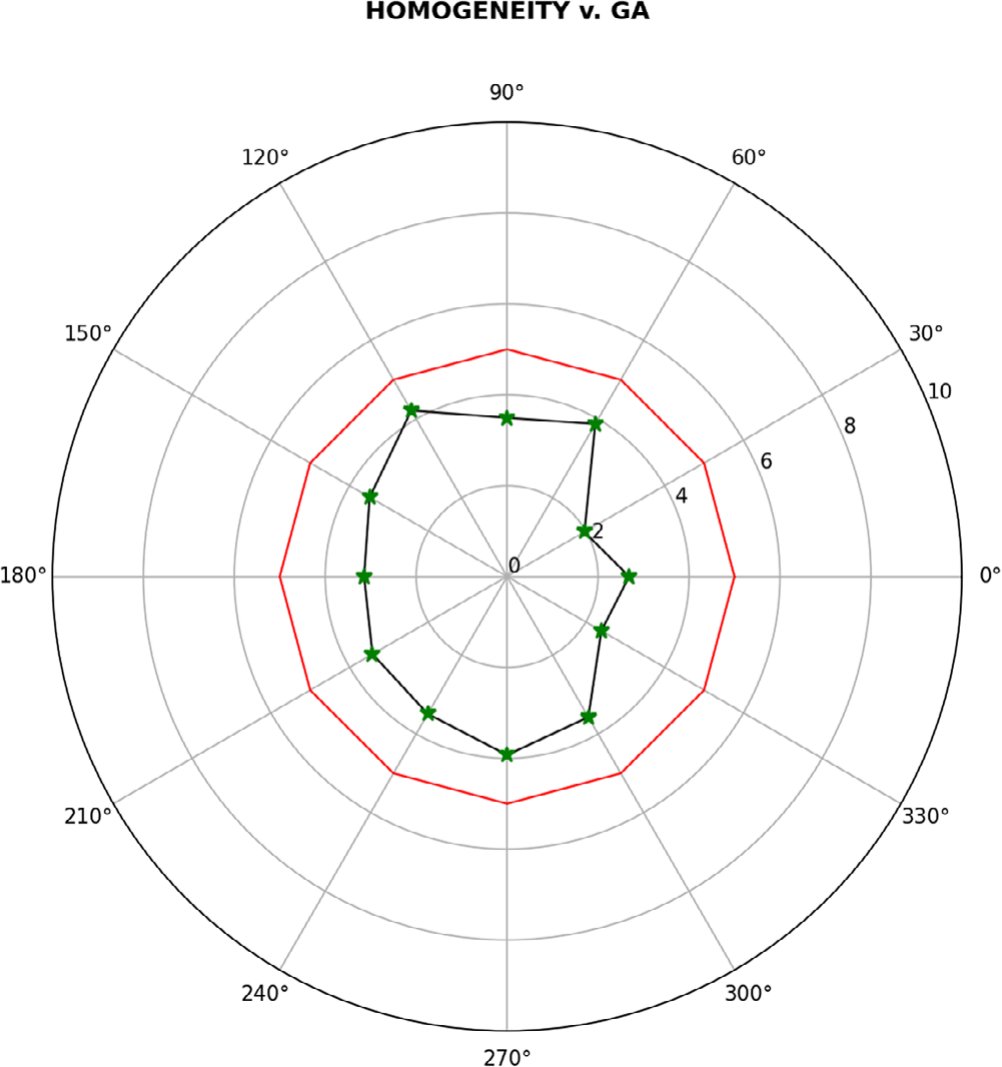
Polar plot of homogeneity as a function of gantry angle (GA), angular variation of field uniformity across all measured orientations.

The polar representation of magnetic field homogeneity as a function of gantry angle demonstrated a clear angular dependence, which confirmed that field uniformity measurements showed different results depending on the specific measurement direction as in Figure 6. The red contour shows the acceptance level of 5 parts per million. The polar plot revealed specific gantry angles that produced higher or lower homogeneity results that matched the expected behavior of nonuniform fields close to the rotational axis.

All measured gantry orientations produced center frequency values that remained tightly clustered with only slight changes, which showed that resonance positions remained constant during the entire acquisition process. The full width at FWHM values showed measurable changes, which depended on the specific gantry angle used. The frequency‐domain results display FWHM values in Hertz and parts per million along with center frequency values, which change according to gantry angle, to show how spectral linewidth changes across different angles.

## 4 DISCUSSION

The Technical Note manuscript presents complete desktop workflow applications that test MRI coils through automated operation while combining image‐based and frequency‐domain quality assessment methods into one unified system. The system achieves better reproducibility between different sessions through its automated processes. It handles dataset ingestion, slice selection, phantom localization, and ROI placement and metrices extraction; this process reduces the need for operator involvement and subjective bias.^8^, ^9^

The image‐based quality assurance process used fixed‐geometry regions together with automated slice selection to produce identical signal statistics results, SNR, and PIU measurement across different analysis sessions. The consistency of the data allows direct comparison between quality assurance metrics from different sessions without requiring manual post‐processing. This process reduces analytic variability that can hide minor performance differences.^1^, ^4^, ^13^, ^14^

The workflows enable users to evaluate multi‐element coils through their automated analysis and handle both torso and head‐and‐neck coil configurations while providing a standardized framework for evaluating combined coil views and individual coil elements. The element‐level metrices provided insights into performance differences across specific areas, which were not visible through only using global performance metrices. It allowed for early detection of channel‐specific performance changes.^6^, ^13^, ^15^ The standardization of processing allows comparison of QA performed at different sessions and coil configurations.^8^, ^9^

The frequency‐domain analysis process generated an objective measurement of magnetic field uniformity through its ability to automatically extract center frequency and spectral linewidth from raw free‐induction decay data. The spectral linewidth exhibits an angular dependence that changes with different gantry positions; therefore, multi‐orientation assessment is essential for understanding field behavior that remains hidden during single‐orientation measurements.^16^ Moreover, this automated workflow is specifically compatible with the quality assurance recommendations of MR simulators described in AAPM Task Group Report 284. The tool allows the system to monitor SNR, PIU and B0 magnetic field homogeneity on the map over time, ensuring that MR‐guided radiotherapy systems have the rigid geometric and imaging tolerances needed to support effective treatment planning and delivery.

To conclude, the current implementation was developed for and validated on a single ViewRay MRIdian 0.35T MR linac system. The coil element configurations include the 12 element torso and 10 element head‐and‐neck, and DICOM private tags for coil identification such as 0051,100F are specific to this platform. Even though the underlying framework utilizing NEMA based SNR computation, Otsu thresholding phantom detection, and fixed geometry ROI placement is platform independent and can generalize traditional diagnostic MRI systems, conventional systems lack gantry dependent acquisitions. However, the parts per million frequency domain workflows for assessing magnetic field homogeneity can be successfully achieved on all MR linac platforms that operate with rotating gantry angles. Consequently, adapting this diagnostic MRI workflow to conventional systems, which can be restricted to single orientation homogeneity analyses, or other MR linac platforms would require altering the acquisition protocols, modifying the coil classification logic, and adjusting analysis outputs to accommodate system specific configurations and validate against the respective vendor acceptance specifications.

## 5 CONCLUSION

We introduce an automated system to perform some of the routine MRI quality control through standardized image assessment and frequency‐domain magnetic field testing. It provides a unified desktop software solution. The workflow enhances reproducibility through its design. It needs less operator work while maintaining uniform analysis procedures and reporting methods, which enables dependable long‐term quality assurance testing in clinical MRI operations.

## AUTHOR CONTRIBUTIONS

**Bhudatt Paliwal**: Conceived and designed the study methodology. Contributed to technical validation, data acquisition, results interpretation, manuscript preparation, and revision. Serves as the corresponding author. **Aviral Bal**: Developed and programmed the automated workflows for weekly QA and for body, torso, and head‐and‐neck coil analyses. **Lokesh Kodali**: Developed the magnetic field homogeneity workflow. Integrated all workflows into a standalone executable application. Prepared documentation and drafted the manuscript. **Dinesh Tewatia**: Performed technical validation and critically revised the manuscript. **Idarto Tan**: Conducted data acquisition and contributed to the interpretation of results

## CONFLICT OF INTEREST STATEMENT

The authors declare no conflicts of interest.

## ETHICS STATEMENT

This study does not involve patient subjects.

## Data Availability

The data and software used in this study are available from the corresponding author upon reasonable request according to the institution's terms and policy.
